# Mixed-Matrix Organo-Silica–Hydrotalcite Membrane for CO_2_ Separation Part 1: Synthesis and Analytical Description

**DOI:** 10.3390/membranes14080170

**Published:** 2024-08-06

**Authors:** Lucas Bünger, Krassimir Garbev, Angela Ullrich, Peter Stemmermann, Dieter Stapf

**Affiliations:** Institute for Technical Chemistry, Karlsruhe Institute of Technology, 76344 Karlsruhe, Germany; angela.ullrich@kit.edu (A.U.); peter.stemmermann@kit.edu (P.S.); dieter.stapf@kit.edu (D.S.)

**Keywords:** hydrotalcite, BTESE gel, delamination, CO_2_ separation, mixed-matrix membrane

## Abstract

Hydrotalcite exhibits the capability to adsorb CO_2_ at elevated temperatures. High surface area and favorable coating properties are essential to harness its potential for practical applications. Stable alcohol-based dispersions are needed for thin film applications of mixed membranes containing hydrotalcite. Currently, producing such dispersions without the need for delamination and dispersing agents is a challenging task. This work introduces, for the first time, a manufacturing approach to overcoming the drawbacks mentioned above. It includes a synthesis of hydrotalcite nanoparticles, followed by agent-free delamination of their layers and final dispersion into alcohol without dispersing agents. Further, the hydrotalcite-derived sorption agent is dispersed in a matrix based on organo-silica gels derived from 1,2-bis(triethoxysilyl)ethane (BTESE). The analytical results indicate that the interconnection between hydrotalcite and BTESE-derived gel occurs via forming a strong hydrogen bonding system between the interlayer species (OH groups, CO_3_^2−^) of hydrotalcite and oxygen and silanol active gel centers. These findings lay the foundation for applications involving incorporating hydrotalcite-like compounds into silica matrices, ultimately enabling the development of materials with exceptional mass transfer properties. In part 2 of this study, the gas separation performance of the organo-silica and the hydrotalcite-like materials and their combined form will be investigated.

## 1. Introduction

Gas separation processes play an important part in reaching carbon-neutral production. Especially high-temperature applications that depend on suitable materials that can efficiently capture CO_2_ above 200 °C receive more attention [1]. Fortunately, sorbent research has made tremendous efforts in the past to investigate the adsorption properties of inorganic materials such as Hydrotalcite-like compounds (HTlcs).

The mineral hydrotalcite, [Mg_6_Al_2_(OH)_16_](CO_3_)(H_2_O)_4_, was first described in 1842 by Hochstetter in serpentine–magnesite rocks in Snarum, Buskerud, Norway [2]. It belongs to the hydrotalcite supergroup of minerals, also known as layered double hydroxides (LDHs). More than 40 structurally and chemically related varieties are known so far [3]. All LDH crystal structures share the similarity of being built up of positively charged brucite-type layers. Their octahedral sites are mainly occupied by M^2+^ and M^3+^ cations, where M^2+^ are Mg, Fe, Mn, Ni, Cu, Ca, and Zn, whereas M^3+^ are Al, Fe, Mn, Co, and Cr. The octahedral layers alternate with negatively charged interlayers occupied by (CO_3_)^2−^, H_2_O, Cl^-^, etc. The crystal structures of hydrotalcite-like compounds and their polytypes have been the object of numerous studies: Allmann and Jepsen (1969) [4] and Bellotto et al. (1996) [5] for the variety quintinite (M^2+^/M^3+^ = 2) and Zhitova et al. (2019) for the “true” hydrotalcite (M^2+^/M^3+^ = 3) [6].

Synthetic materials on an LDH basis are in great demand due to their useful physicochemical properties [7]. Their ability to function as a high-temperature adsorption agent for CO_2_ has been studied extensively [8,9,10,11]. However, the bulk of these investigations focuses on HTlc materials in their layered and powdery forms rather than technical applications. To bridge this gap and realize their potential in real-world applications, a transformation of HTlc is essential. The preferred approach involves converting them into a delaminated state, which offers an enlarged surface area for more effective interactions [12]. Delaminated HTlcs have a broad range of applications, including their use as catalysts [13], enhanced drug delivery [14], biosensors, and adsorbents [15]. Their use as pure material membranes has not shown convincing results due to the resulting porosity that facilitates only Knudsen diffusion [16,17]. However, their utilization as adsorptive mass transfer agents in polymeric membranes has already demonstrated efficacy at lower temperatures [18].

For high-temperature membrane applications, a mixed-matrix approach seems to be promising. Attempts to incorporate hydrotalcite-derived substances in water-based γ-alumina matrices yielded mesoporous microstructures with unselective Knudsen diffusion [19]. Amorphous organo-silica has proven to be a suitable material as it forms a microporous microstructure that enables selective mass transport and withstands harsh process conditions [20,21,22,23]. These silica matrices are prepared from alcohol-based dispersions, which are highly sensitive to water. In the preparation of the mixed-matrix membrane, the hydrotalcite-derived adsorption agent must, therefore, be in a stable alcohol-based form, completely devoid of water content, while possessing similar drying characteristics to the matrix. Gels derived from 1,2-bis(triethoxysilyl)ethane (BTESE) are considered suitable matrices as their linking units could be used to control the membrane pore sizes. However, these membranes experience a loss of selectivity at higher temperatures due to a decrease in adsorptive affinity for CO_2_ [24,25] and an increase in N_2_ permeance caused by activated permeation [26,27,28]. An excellent review of the state of the scientific knowledge and technology regarding the application of organo-silica membranes for gas separation purposes is given by I. Agirre et al. [29]. The high-temperature adsorption agent HTlc combined with a microporous organo-silica xerogel matrix is envisioned to offset their respective weaknesses.

The use of delaminating and dispersing agents presents challenges as they tend to persist on the substance post-drying. While these agents can be eliminated during thermal treatment, they create porosity, thereby compromising the selectivity of the resulting membrane for gas separation applications.

Successful delamination processes using formamide as a solvent have shown promising results; however, there is an effort to find alternatives due to its toxicity. Recent research suggests positive outcomes with a combination of dodecyl sulfate and n-butanol as an alcoholic dispersant [30]. Additionally, experiments with sodium acetate have demonstrated stable HTlc dispersions in ethanol [31]. However, all these processes combine the disadvantage of either utilizing large amounts of dispersing agents, a solvent with different drying properties than ethanol, or the HTlc not being in its active CO_2_ adsorptive form. The synthesis of the precursor for the CO_2_ adsorbing compound, hydrotalcite, offers various routes described in the literature [32]. However, for the specific application of creating stable ethanol-based dispersions, the focus lies particularly on preparing particles of a nanometer size.

Therefore, this study shows an approach to creating an HTlc-derived adsorption agent in ethanol without the use of a dispersing agent. Firstly, the preparation method of the hydrotalcite is presented, then its delamination in water, and finally, the transformation of the calcined active form into the ready-for-use ethanol dispersion. Furthermore, the combination of the produced dispersion is investigated after mixing it with the aforementioned silica matrix based on BTESE gel derivates. All described steps are analyzed using various techniques to determine their respective composition and structural properties and how they vary during processing.

## 2. Materials and Methods

### 2.1. Preparation

#### 2.1.1. Hydrotalcite

The synthesis of hydrotalcite is adapted from the work of Gardner et al. [33,34]. To control the particle size, water is changed to methanol as a solvent. In 100 mL methanol, 6.1 g of magnesium chloride hexahydrate and 2.41 g of aluminum chloride hexahydrate, with a molar ratio of Mg/Al = 3, are dissolved. This mixture is heated to 65 °C and stirred under reflux. To start the LDH-forming reaction, 3.8 g of sodium hydroxide is dissolved in 100 mL of methanol and added dropwise to the metal salt solution. For a small particle size distribution, the synthesis time from Gardner’s work is reduced from 3 days to 1 h. The synthesized product is rigorously washed with methanol to dissolve the by-product NaCl and subsequently dried at 50 °C. The resulting sample is further referred to as HTlc-pure (1) throughout the manuscript.

After allowing the reaction mixture to reach room temperature, it is subjected to centrifugation, and the resulting pellet is dispersed in water. This procedure is repeated until the pH level reaches neutrality. The dispersed material is then left in water overnight to undergo delamination, resulting in a transparent dispersion. This specific form of the delaminated substance is subsequently dried and referred to as HTlc-delaminated (2a). The membrane coating process yields structures with oriented platelets, as seen in the SEM images in part two [1]. To investigate the influence of this orientation, oriented samples are produced by dropping single droplets of the transparent dispersion onto a sample carrier. This process is repeated several times, resulting in platelets that are oriented parallel to the carrier surface. Those samples are further referred to as HTlc-delaminated-oriented (2b).

The delaminated dispersion is subjected to thermal treatment at 400 °C for 3 h in air to activate the agent. Following this treatment, the resulting powder is placed in an evacuated desiccator until the subsequent analysis. This calcined form is labeled as HTlc-calcined (3).

#### 2.1.2. Organo-Silica Matrix

For the synthesis of the matrix material, which enables CO_2_ separation, the approach is based on the work of Van Gestel [22]. He based the synthesis on pioneering work conducted at the University of Twente in the group of Ten Elshof [20]. The synthesis method used in this work is a simplified version. It involves mixing 16.655 mL of the membrane-forming precursor (BTESE) with 28.14 mL of ethanol (both thermo scientific chemicals), 0.63 mL of nitric acid (65 wt%), and 4.57 mL of water. Respective amounts result in a water-to-hydrolyzable ethoxy group ratio of one. No ice bath is used due to the rapid mixing of the mixture. The resulting mixture is heated to 60 °C and refluxed for 90 min, followed by cooling to room temperature. To stabilize the sol for storage, an additional 50 mL of ethanol is added, and the mixture is stored in the refrigerator (stock sol). To produce the gels, the stock sol was dried at room temperature to produce the lyogel (BTESE-derived lyogel). Additional thermal treatment was carried out at 300 °C for 3 h under an N_2_ atmosphere to produce the xerogel. This xerogel is further referred to as BTESE-derived xerogel and is also analyzed for comparison.

#### 2.1.3. Mixed-Matrix

To mix the HTlc with the organo-silica, the HTlc must first be converted into an ethanol-based dispersion. Two grams of the calcined and ground Htlc (3) were mixed with 50 mL of ethanol and redispersed using an ultrasonic device for 20 min. Afterward, the dispersion was centrifuged for 20 min at 4000 rpm to remove any undispersed particles. This process was repeated until no undispersed particles were found and a stable dispersion was formed. Then, 5 mL of this sol was mixed with 5 mL of the organo-silica stock-sol and filled up with 40 mL of ethanol. Subsequently, this sol was dried into a powder for analysis. This form is denoted as HTlc-modified organo-silica (4) and the final form for the high-temperature membrane application.

The membrane-forming process and resulting SEM images of the prepared membranes and schematic drawings of the microstructure are given in part 2 of this publication [1].

### 2.2. Analytical Methods

Structural information is obtained by a combination of infrared (IR) and Raman spectroscopy and X-ray diffraction (XRD). Additionally, its thermal behavior is investigated by thermogravimetric (TGA) experiments.

#### 2.2.1. X-ray Diffraction (XRD)

X-ray diffraction measurements were conducted on an Empyrean diffractometer (Malvern-PANalytical, Almelo, The Netherlands) with a Cu-Kα tube operated at 40 mA and 40 kV equipped with a multistrip PIXcel3D detector (255 channels, simultaneously covering 3.347° 2θ) and a Bragg–Brentano HD device. The powder patterns were measured in the range of 5 to 90° 2θ with step size 0.013° 2θ, time per step 1s, slits of 0.125°, soller slits of 2.3°, and sample rotation. The powder patterns were evaluated with HighScorePlus v.5.1 software. The phases were identified using the ICDD (2004) and COD (2021) databases.

Rietveld refinements were performed with TOPAS v.7 for the estimation of the unit cell parameters and the size of coherent scattering domains of hydrotalcite. The structure data of Zhitova et al. [5], ICSD Nr.-133744 and ICSD Nr.-133742 have been used as a starting model.

#### 2.2.2. IR Analysis

IR measurements in Attenuated Total Reflection (ATR) arrangement were performed on powder samples in a Golden Gate ATR cell with a diamond crystal (Specac LTD, Orpington, UK) positioned in a Tensor II spectrometer (Bruker Optics, Ettlingen, Germany) equipped with a deuterated triglycine sulfate (DTGS) detector. Spectra with 64 scans and a spectral resolution of 2 cm^−1^ in the range of 400–4000 cm^−1^ were acquired.

#### 2.2.3. Raman Spectroscopy (RS)

Raman spectroscopic analyses were performed on the powdered samples dusted on glass slides. A WITec alpha300 R equipped with a UHTS300 spectrometer (300 mm focal length) and a Zeiss confocal microscope were employed for single-spot measurements. As an excitation source, a 533 nm laser operated at 50 mW (on sample) was used. The measurements were performed with a 100× objective with a numerical aperture of 0.9 using 600 and 1800 groves/mm holographic gratings with spectral resolution better than 3 cm^−1^ and 1 cm^−1^, respectively. A high-performance back-illuminated CCD camera with 96% quantum efficiency was used for detection. Typical acquisition times were between 5 and 20 s with 5–10 scans. The phase identification was aided by the RRUFF mineral database and an own database integrated into the WITec True Match program. The data processing and preparation of the Raman images were performed with the Project 5.3+ software from WITec (Ulm, Germany). The single spectra were corrected by the cosmic ray removal algorithm, and the consequent subtraction of the background was calculated using the shape algorithm.

#### 2.2.4. Thermal Analysis (TA)

The thermal analyses (TA) were performed on an STA 403 Jupiter F3 (Netzsch, Selb, Germany) equipped with a Pt oven, coupled to a Tensor 27 Fourier transform infrared (FTIR) spectrometer (Bruker-Optics, Ettlingen, Germany). The TA device was purged with N_2_ (2 × 60 mL/min) and was connected via a transfer gas line to an FTIR gas cell (both kept at 200 °C to prevent condensation), where the detection of the gases released upon heating was performed with a liquid nitrogen cooled MCT (mercury cadmium telluride) detector. The samples were heated at a rate of 10 K/min, and IR spectra of released gases were acquired with 32 scans and spectral resolution of 4 cm^−1^ in the range of 600–4000 cm^−1^. The calibration of the FTIR spectrometer was carried out with certified test gases in the validity range of Beer–Lambert’s law. The quantification of H_2_O and CO_2_ was performed using the method developed by Merz et al. [35].

## 3. Results and Discussion

### 3.1. XRD Results

**Figure 1:**
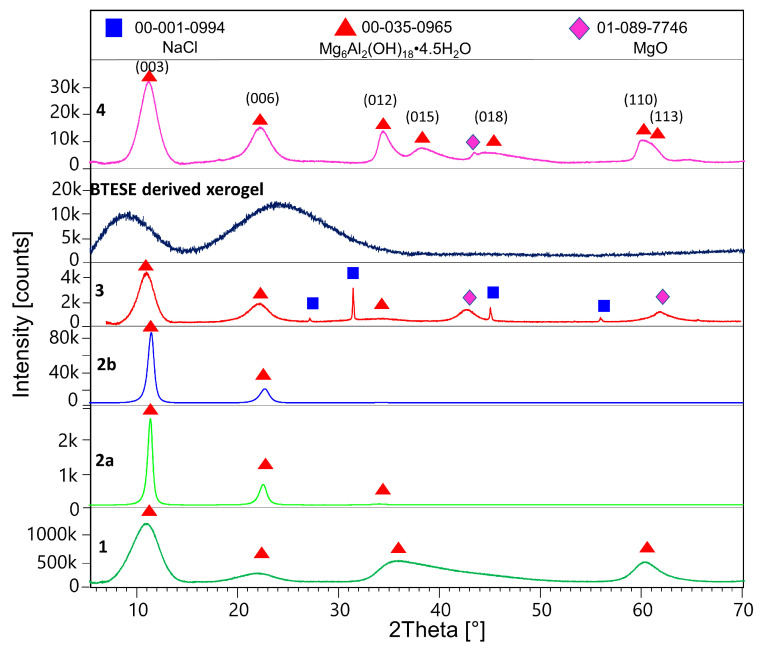
Powder XRD patterns of the methanol-washed HTlc-pure sample (1), HTlc-delaminated hydrotalcite (2a), HTlc-oriented sample (2b), HTlc-calcined sample (3), BTESE-derived xerogel, and HTlc-modified organo-silica (4).

Figure 1 shows the X-ray powder patterns of samples 1, 2a, 2b, 3, BTESE, and 4. Table 1 shows refined unit cell parameters considering the 3R and 2H polytypes and calculated sizes of the coherent scattering domains (results from the double Voigt approach) for hydrotalcite in the corresponding samples.

After being washed with methanol, the synthesized hydrotalcite HTlc-pure shows very broad reflections pointing to a very small size of the coherent scattering domains of about 1.6 nm (further referred to as crystal size). This confirms the findings of Gardner et al. [32] pointing to a tiny crystal size of hydrotalcite precipitated from methanol solutions of Mg and Al salts. A crystal size of 1.6 nm can also suggest a thickness of one unit cell parameter in the z direction of the 2H polytype consisting of two brucite layers (Table 1). The delaminated and oriented samples (2a and 2b), on the other hand, show very sharp reflections, pointing to crystal sizes of 10.6 and 8.4 nm, respectively. The significantly higher values for samples 2a and 2b are expected, given the complete hydrolysis of the methoxide ions and the stacking of the hydrotalcite platelets along the [001] direction after drying. The term “delamination” refers to the fact that upon drying, although almost perfect stacking along the z direction of hydrotalcite takes place, a strong disorder in the xy plane is observed. Therefore, only 00l reflections are seen in the patterns of samples 2a and 2b. A tendency for decreasing the size of the *c*-unit cell parameter from 25.2 to 23.68 to 23.56 Å is also observed for samples 1, 2a, and 2b, respectively. This is consistent with the expected exchange of methoxide with H_2_O. The largest c parameter of sample 1 proves the intercalation of the alkoxide anions between the brucite layers [32]. Samples 2a and 2b show the typical unit cell parameters for H_2_O exchanged hydrotalcites (basal spacing of 003 = 7.9Å). The calcined sample (3) once again shows broad reflections of hydrotalcite due to the expected disorder accompanied by additional broad peaks at around 43 and 62 °2Theta, which could be assigned to MgO (ICDD: 01-089-7746). The *c*-unit cell parameter shows a small increase to 24.22 Å. The crystal size decreases to 3.2 nm. Therefore, the calcination at 400 °C leads to partly dehydrated, disordered “hydrotalcite” with similar crystal features as the starting material with disrupted long-range order. In addition, the sample shows some tiny impurities of NaCl, which is a by-product of the synthesis of hydrotalcite. BTESE organo-silica gel shows two broad “humps” at about 8 and 24 °2Theta. The HTlc-modified organo-silica membrane sample (4) shows similar features as sample 3 in its powder pattern but is accompanied by additional 0 kl and 11 l reflections, thus witnessing a random orientation of the “hydrotalcite” platelets which seem to be homogenously distributed in the gel matrix. Considering the crystal size of samples 3 and 4, they consist of four brucite layers.

### 3.2. IR Spectroscopy

**Figure 2:**
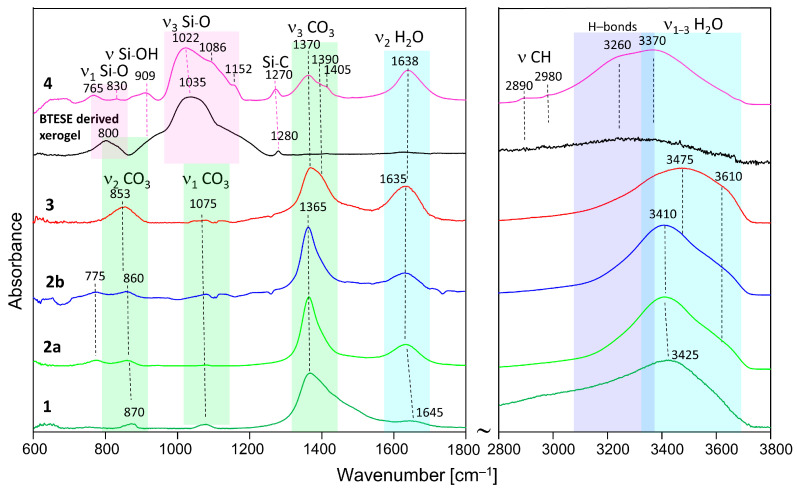
ATR-IR spectra of methanol-washed hydrotalcite (HTlc-pure: 1), HTlc-delaminated (2a), HTlc-delaminated-oriented (2b), HTlc-calcined hydrotalcite (3), BTESE-derived xerogel, and HTlc-modified organo-silica (4).

The IR spectra are shown in Figure 2, and relevant bands are summarized in Table 2. Spectrum 1 and 2a are hydrotalcite synthesized and washed with methanol and the delaminated sample, respectively. The assignment is made based on the results of Kloprogge et al. [36] and Frost et al. [37], as well as [38,39,40]. Sample 1 shows broader bands than 2a and 2b, which is consistent with the XRD results. The main band situated at 1365 cm^−1^ is due to the asymmetrical ν_3_ (CO_3_) mode. The broad high-frequency slope is due to the absorption of ν_2_ CH_3_ bending modes, which are expected in the range of 1440–1480 cm^−1^ [41], and the possible interaction of the CO_3_ groups of methanol [42]. Additionally, it may witness a large ∆ν_3_ CO_3_ splitting typical for adsorbed CO_2_ as monodentate or bidentate carbonate [38]. The corresponding ν_2_ (CO_3_) and ν_1_ (CO_3_) modes are seen at about 870 cm^−1^ and 1075 cm^−1^, respectively. The relatively high intensity of the latter band in the spectrum of sample 1 indicates that this carbonate is due to monodetate and bidentate adsorption of CO_2_ [38]. It possibly shows contributions of C-O stretching from CO2CH_3_OH (in the range of 1020–1060 cm^−1^), which is known to be sensitive to interactions with hydrogen bonding [39].

The broad band at 1645 cm^−1^ belongs to the bending vibration of H_2_O. The broad absorption is possibly due to Mg(Al)-OH interactions. The high-frequency region, 2800–3800 cm^−1^, is marked by a broad band due to ν_1_ OH (centered at 3425 cm^−1^) and a very broad low-frequency slope due to ν_1_ CH_3_ and its interaction with OH. Thus, the presence of methoxide anions is once again confirmed.

Samples 2a and 2b show quite similar spectra with bands at 775 cm^−1^ (translation Al-OH) and 860 cm^−1^ (ν_2_ CO_3_). The strongest band is once again the ν_3_ CO_3_ (1365 cm^−1^), lacking the contribution of CH_3_ bending modes. It may also indicate smaller ∆ν_3_ CO_3_ splitting due to the presence of polydentate CO_3_ tightly bonded in the hydrotalcite structure. The band at 1075 cm^−1^ (ν_1_ CO_3_) also lacks the contribution of CO stretching of methanol. The delamination with H_2_O also leads to well-pronounced H_2_O bands at 1635 cm^−1^ (bending) and 3410 cm^−1^ (ν_1_ OH in H_2_O) with a shoulder at 3610 cm^−1^ (ν_1_ OH). The presence of the OH bands at 775 cm^−1^ highlights the difference between sample 1 on one side and samples 2a and 2b on the other side.

H_2_O interacting with interlayer carbonate in the hydrotalcite structure gives rise to increased absorption between 3000 and 3400 cm^−1^.

The HTlc-calcined sample (3) shows, once again, very broad bands due to increased disorder. The ν_2_ bending band of CO_3_ shows a shift to a lower frequency (853 cm^−1^), whereas the asymmetrical stretching band ν_3_ (CO_3_) shifts to a higher frequency [1370 cm^−1^]. The broader ν_3_ CO_3_ band (with a shoulder at 1390 cm^−1^) may also indicate the presence of different environments of carbonate having on one side polydentate character but also monodentate and bidentate surface carbonate on the other side. Similar behavior is observed for the OH stretching bands (shift to 3475 cm^−1^), pointing to the presence of more slightly bonded hydroxyls as a result of rehydration.

The IR spectrum of the organo-silica xerogel BTESE is characterized by strong and broad multiple bands in the range of 950–1200 cm^−1^ with a maximum intensity located at about 1035 cm^−1^. These bands could be attributed to asymmetric Si-O stretching modes (ν_3_ Si-O) [26] in ring-like siloxane moieties formed via the condensation reaction of silanol groups. Compared to pure SiO_2_ gels (TEOS, Aerosil), which show frequencies centered at about 1070–1100 cm^−1^ [43], the lower frequency in BTESE could be explained by lower silicate polymerization induced by the presence of organic moieties in the silica network [44,45]. Additionally, it may be due to the lower rate of hydrolysis, as the synthesis was performed with low H_2_O content to keep the particle size as low as possible. The band centered around 800 cm^−1^ is characteristic of symmetric Si-O stretching (ν_1_ Si-O). A broad absorption seen as a shoulder at about 910 cm^−1^ is ascribed to the stretching vibration of silanol bonds (Si–OH). Its intensity is rather low, which is expected in fired xerogels where enhanced condensation has taken place. The band at 1280 cm^−1^ is characteristic of Si-C stretching vibrations [46]. The range of 2800–3800 cm^−1^ is dominated by broad bands due to ν_1_ and ν_3_ O-H symmetric and asymmetric stretching of H_2_O and OH groups. Due to the firing at 300 °C, a very low intensity of H_2_O and OH-related bands is observed. In addition, the low-intensity bands at 2890 and 2980 cm^−1^, typical for C-H stretching vibrations, prove the presence of CH_2_ and CH_3_ in the Si–(CH_2_)_2_–Si and (–OCH_2_CH_3_) groups, respectively. The low intensity of the C-H bands marks a high degree of hydrolysis and consequent condensation of silanol groups to siloxanes.

The spectrum of the HTlc-modified organo-silica membrane sample (4) retains similar features characteristic for both BTESE and HTlc-calcined hydrotalcite (3) but with some frequency shifts of the original bands. For example, the organo-silica rings seem to be strongly influenced by the presence of hydrotalcite fragments showing shifts of ν_1_ Si-O from 1035 to 1022 cm^−1^ and of Si-C from 1280 to 1270 cm^−1^. The band at 800 cm^−1^ in BTESE is also shifted, pointing to a change in the Si-O-Si bridging angle. The spectrum of sample 4 shows more pronounced silanol groups (909 cm^−1^). The formation of silanol groups correlates with indications for a strong hydrogen-bonded system, which is manifested by a low-frequency shift of the stretching vibrations of H_2_O and OH in the spectrum of sample 4 compared to the rest of the samples. The silanol band frequency is higher than that reported by Moriyama et al. for pure BTESE gels (890 cm^−1^) [26]. These observations imply that the interaction between hydrotalcite and BTESE gel most probably takes place via enhanced hydrogen bonding between OH and H_2_O of hydrotalcite and Si-O-Si, SiOH, and Si-CH_2_(_3_) moieties of BTESE gel. The CO_3_ modes clearly show the presence of two bands at 1370 and 1405 cm^−1^ with ∆ν_3_ splittings once again higher (35 cm^−1^) than that observed for the calcined sample (20 cm^−1^). It possibly shows an interaction between the surface CO_3_ groups of hydrotalcite and the xerogel.

### 3.3. Raman Spectroscopy

**Figure 3:**
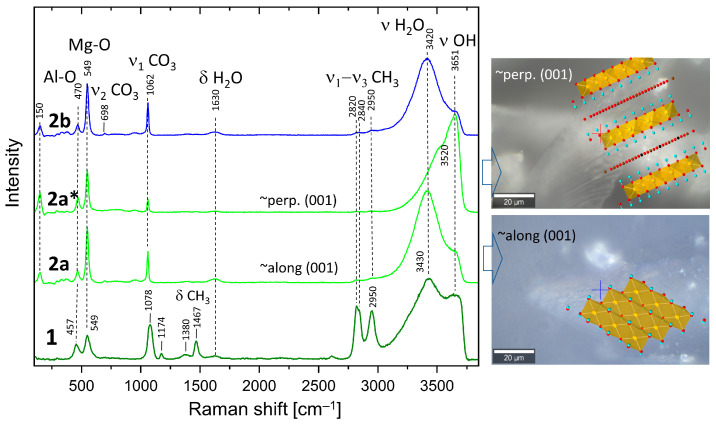
Raman spectra of synthesized and washed HTlc-pure sample (1), HTlc-delaminated (2a, 2a*), and HTlc-oriented hydrotalcite (2b). Spectrum 2a of the delaminated sample is taken almost along the [001] direction, thus enhancing the H_2_O bending (1630 cm^−1^) and stretching bands (3420 cm^−1^), whereas the spectrum 2a* is measured along the octahedral layers. As a result, the intensity of the OH bands (3651 cm^−1^) is enhanced. The optical images overlaid with the corresponding approximate orientations of the hydrotalcite structure are shown on the right-hand side. Crosses mark the exact positions of the Raman measurements. Completely oriented sample (2b) shows a similar spectrum to 2a (Red dots for oxygen atoms and blue dots for hydrogen atoms).

The Raman spectra of the 1, 2a, and 2b samples are presented in Figure 3. All samples show bands at 549 cm^−1^ and 457 cm^−1^, characteristic for hydrotalcite, which could be assigned to stretching and bending vibrations (*M*–O, *M*–O–*M*, and O–*M*–O) within the octahedral layers [36]. Most probably, the band at a lower frequency is due to Al-O vibrations, whereas the band at 549 cm^−1^ could be assigned to Mg-O due to the difference in the atomic weight and corresponding quantities. These bands are sharper in samples 2a and 2b. Very pronounced is the band at 1078 cm^−1^ (sample 1) assigned to the symmetrical stretching vibration of CO_3_ (ν_1_ CO_3_). The corresponding band in samples 1b and 2 is much sharper and shows a frequency shift to 1062 cm^−1^. Additionally, the spectrum of sample 1 shows the presence of bands at 1467 cm^−1^ and 2820 cm^−1^, 2840 cm^−1^, and 2950 cm^−1^ belonging to bending and ν_1_ and ν_3_ stretching of CH_3_ groups in methoxide anions [47,48], respectively. Therefore, it is evident that methoxide anions (OR) are grafted to the brucite layers, partially exchanging OH groups after the formula [M^2+^_(1−x)_ M^3+^_x_ (OH)_2−y_] (OR)_y_] A^n−^_(x/n)_ (OR)_y_ .mH_2_O [32], where A^n−^ represents additional anions like CO_3_^2−^, which are inevitably present due to the contact with CO_2_ from the air.

Low-intensity bands at 1174 and 1380 cm^−1^ are also due to methanol. Broad bands belonging to bending and stretching modes of H_2_O and OH groups are present at 1630 cm^−1^ (δ H_2_O), 3430 cm^−1^ (ν_1_/ν_3_ H_2_O), and 3650 cm^−1^ (ν_3_ OH). Spectra marked as 2a and 2a* are taken in two orientations of the delaminated sample. The former is taken almost along the [001] direction, leading to an enhanced H_2_O stretching band (3420 cm^−1^) compared to that taken along the octahedral layers (perpendicular to [001]). Spectrum 2a* shows enhanced intensity of the OH bands (3651 cm^−1^) due to the larger vector components of the OH bonds seen in this direction. The right-hand side of Figure 3 shows optical images overlaid with the corresponding approximate orientations of the hydrotalcite structure. The band at 150 cm^−1^ also shows a higher intensity in the spectrum of sample 2a*, which implies that it may also be due to an OH mode. The opposite trends are observed for both Mg-O modes (549 cm^−1^), CO_3_ bands (ν_1_-CO_3_), and δ-H_2_O, which is consistent with the proposed orientation of the crystals. The spectrum of the oriented sample (2b) is strikingly similar to that of 2a, which confirms these considerations once again. There are remarkable differences in the frequencies of Al-O and CO_3_ bands in the methanol-washed sample (1) and samples 2a and 2b. Whereas the former shifts to higher frequency (470 cm^−1^), the latter shifts to 1062 cm^−1^. Therefore, methanol strongly influences the carbonate groups in the interlayer and possibly selectively the Al from the octahedral layers.

**Figure 4:**
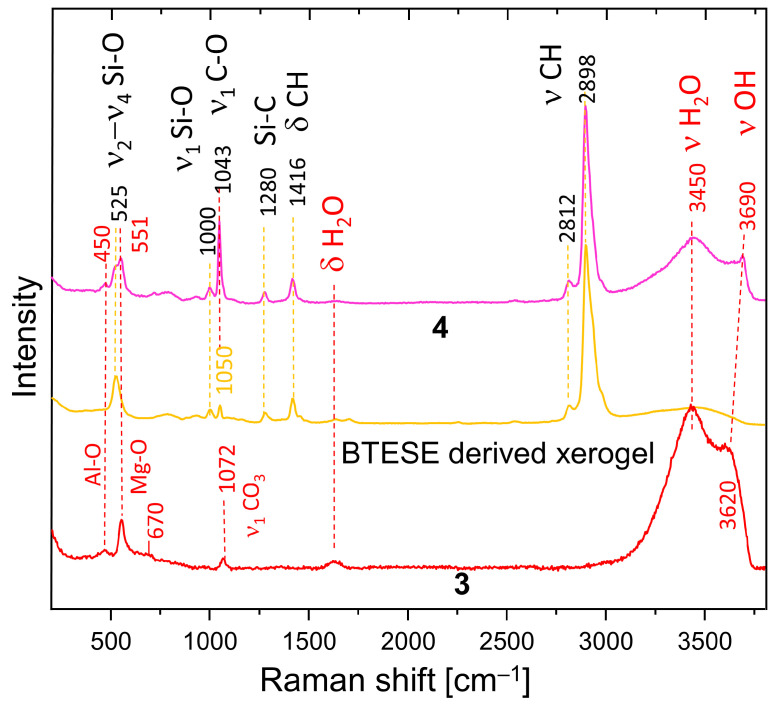
Raman spectra of HTlc-calcined sample (3), BTESE xerogel, and HTlc-modified organo-silica (4).

Figure 4 shows a comparison of the Raman spectra of HTlc-calcined hydrotalcite (3), BTESE, and mixed HTlc-modified organo-silica membrane (4). Sample 3 shows broader bands than the delaminated and oriented samples. The frequencies of the octahedral Mg-O vibrations change to a minimally higher frequency (551 cm^−1^), whereas those typical for Al-O shift to the lower (450 cm^−1^). The most intense band of MgO is expected also in this region [49]. Additionally, a broad band occurs centered at about 670 cm^−1^, possibly due to the partial formation of disordered spinel, which needs to be avoided as it has no relevant CO_2_ adsorption capacity [50].

Both are expected products from the breakdown of hydrotalcite [51]. The symmetrical stretching of CO_3_ is observed at 1072 cm^−1^ instead of 1078 cm^−1^, pointing to a change in the environment of the carbonate anion. In addition, the intensity of this band is considerably lower than in the spectra of not calcined samples (Figure 3). The difference in the vibrational frequencies of the stretching bands of H_2_O and OH in the spectrum of the calcinated sample (3450 cm^−1^, 3620 cm^−1^) compared to the spectra of samples 1b and 2 (3420 cm^−1^, 3650 cm^−1^) also witness a change in the interlayer environment. A partial rehydration of the sample from air moisture seems possible.

BTESE shows typical CH stretching bands in the range of 2800–3000 cm^−1^. They consist of a very intense band at 2898 cm^−1^ with high-frequency shoulders at 2930 and 2980 cm^−1^ and a small band at a lower frequency (2812 cm^−1^). A band at 1416 cm^−1^ manifests the corresponding bending vibrations. A question arises about the difference in the intensity of the C-H stretching bands in the IR and Raman spectra. A possible explanation is that the ethoxide groups in the xerogel are oriented to the centers of the siloxane rings. Therefore, a relatively low number of such groups is observed at the surface, thus becoming “invisible” for ATR measurements. Si-C stretching is observed at 1280 cm^−1^. In addition, bands typical for breathing vibrations of siloxane rings appear at 525 cm^−1^. They are at a higher frequency than the four-membered siloxane rings of the gels derived from TEOS (490 cm^−1^) [52]. Broad bands at about 790 cm^−1^ and 925 cm^−1^ are attributed to Si-O-Si and Si-OH vibrations, respectively. At 1000 cm^−1^, (ν_1_ Si-O) are present. The assignment of the band at 1050 cm^−1^ is somewhat ambiguous. It is also present in non-fired BTESE gels but with lower intensity (not present). Therefore, it could be due to the C-O stretching mode of ethoxy groups H_3_C-O-R on one side or to Si-O stretching modes in environments different than siloxanes (Si-O-Si). These might be the (C)Si-O environments typical for the BTESE rings.

The Raman spectrum of the mixture of BTESE and calcined hydrotalcite (4) shows complementary features characteristic for both constituents and, in addition, strong indications of interactions between the two. The band at 1043 cm^−1^ could be due to C-O modes of methanol used as a dispersion medium or ethanol remnants from the synthesis of the BTESE, but an influence of (C)Si-O, as discussed above, is also possible. The intensity of this band is much higher in spectrum 4 than in spectra 3 and BTESE, respectively.

Therefore, the more probable explanation is that this band is due to adsorbed CO_2_ as a carbonate, which may be shared between BTESE and calcined hydrotalcite. The small shoulder on the high-frequency side of this band shows the presence of additional environments of CO_3_ in the sample, also witnessed by the ∆ν_3_ splitting in the IR spectra. A new feature is the pronounced OH band shifted to 3690 cm^−1^. The higher frequency indicates a lower dependence on the environment, which is characteristic of unassociated OH groups on the surface of the mixture. The unchanged frequencies of the Mg-O and Al-O bands give a hint that the interaction between hydrotalcite and BTESE gel takes place over OH and CO_3_ species characteristic for the interlayer and not over bridging oxygens from the main octahedral layers. Whatever assignment of the band at 1043 cm^−1^ is correct (ethoxy groups or CO_3_ groups), the observed frequency shift and intensity might be an indication for cross-linking between hydrotalcite and BTESE gel.

### 3.4. Thermal Analysis

**Figure 5:**
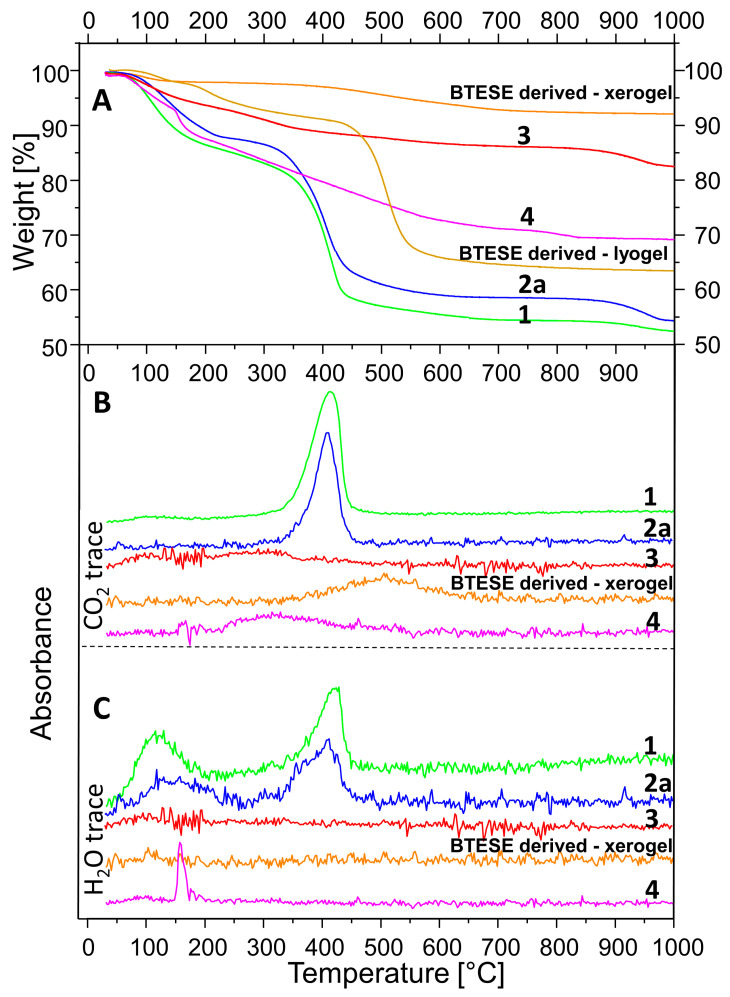
TGA coupled with simultaneous FTIR analysis. Thermogravimetric analysis (**A**); CO_2_ release (**B**); H_2_O release (**C**). Samples: HTlc-pure (1); HTlc-delaminated (2a); HTlc-calcined (3), BTESE-derived xerogel; BTESE-derived lyogel, HTlc-modified organo-silica (4).

The TGA curves are presented in Figure 5A. Figure 5B,C shows the traces of CO_2_ and H_2_O releases, respectively, as a function of temperature. A comparison between A, B, and C allows the assignment of the observed weight loss. Sample 1 shows an early inflection point (at 70 °C) of the weight loss between 30 and 200 °C probably due to two factors: (1) a large quantity of adsorbed H_2_O because of the very high specific area and (2) decomposition of the methoxide anions. Compared to the H_2_O exchanged and delaminated sample (2a), the weight loss in the range of 30–250 °C of sample 1 takes place not only earlier but is also higher: 14.7% compared to 12.2%. The latter correlates with the ideal stoichiometry of hydrotalcite, which considers it a loss of absorbed and interlayer H_2_O. Between 300 and 500 °C, a strong mass loss takes place attributed to almost simultaneous dehydroxylation of the brucite layers and decarbonation of hydrotalcite [53,54]. A comparison of Figure 5B,C for sample 2a allows the interpretation that the H_2_O could be separated into two events, one of which precedes the CO_2_ release marginally.

The observed weight loss between 300 and 500 °C for samples 1 and 2a (27.8% and 26.5%) is way too low compared to the expected theoretical value for full decarbonation and dehydroxylation of hydrotalcite with formula (Mg_6_Al_2_(OH)_16_(CO_3_).4H_2_O): CO_2_ + 8H_2_O = 7.3% + 23.9% = 31.2%, based on an M = 604 g/mol. On the other hand, sample 1 is composed of methoxide instead of pure H_2_O in the interlayer, and the detected carbonate should only be due to a surface reaction with the CO_2_ from the air, as shown by the large ∆ν_3_ shift in the IR spectrum. In addition, this carbonate persists at higher temperatures, as observed in [38]. Therefore, the full decomposition of hydrotalcite is reached only at higher temperatures. The small step observed above 900 °C evidences an additional weight loss, especially considering the TG curves of samples 2a and 3. The total mass loss of samples 1 and 2 is 47.1% and 45.3%, respectively, the latter being very close to the theoretical value of 43.1%.

The higher loss of sample 1 is expected, given the large presence of methoxide groups. Calculation of the molar mass considering full substitution of OH by H_3_CO^1−^, the presence of CO_3_^2−^ and 4H_2_O (M = 693 g/mol) would result in a total mass loss of 50.4 %, which seems to be exaggerated but explains the higher loss of sample 1. Other authors also reported similar observations [49]. A small contribution to the weight loss above 900 °C could be the melting of residual NaCl, but its content is too low to account for the whole effect. It cannot be ruled out that some Cl is bonded in the hydrotalcite interlayer [55] and references therein].

Sample 3 (HTcl-calcined) shows a total mass loss of 17.2%. It is subdivided into three events taking place between room temperature and 200 °C (6%), 200–400 °C (4.9%), 400–700 °C (2.6%), and 700–1000 °C (3.7%). This shows once again that, on the one hand, the complete decomposition of hydrotalcite takes place at higher temperatures, and on the other hand, particular rehydration and recarbonation take place after calcination.

The thermal behavior of the BTESE-derived xero- and lyogel show a minor weight loss due to adsorbed water below 150 °C. The lyogel, which has not been exposed to temperatures above 50 °C, shows condensation reactions above 200 °C, which results in further water loss. The fired xerogel shows no further condensation reactions. At temperatures above 450 °C, the lyogel undergoes degradation and combustion of ethoxide groups and ethylen bridges with C_2_H_4_, C_2_H_6_, and C_2_H_5_OH as products [56]. The total weight loss for the lyogel accumulates to 37%.

The continuous weight loss for the BTESE-derived xerogel between 300 and 700 °C could be attributed to non-reacted degradation and combustion of ethoxide groups (–OCH_2_CH_3_) and ethylene bridges (–CH_2_–CH_2_–). In this range, a release of CO_2_ was detected by the IR spectroscopy (Figure 5B). The total weight loss adds up to 7.6%.

Sample 4, as a combination of (3) and BTESE-derived lyogel, shows a total weight loss of 31%. Up to 150 °C, a 5.3% loss is due to the release of adsorbed H_2_O and possibly ethanol. Between 150 and 200 °C, a sharp weight loss of 6.9% is observed, which corresponds to the water loss from the condensation of the Si–OH groups upon the formation of Si–O–Si bonds. In the range of 200–650 °C, a gradual weight loss takes place, which is mostly due to the release of CO_2_, as seen in Figure 5B. It seems to be a combination of CO_2_ release from hydrotalcite remnants and combustion of ethoxides, as described above. The sharp loss between 150 and 200 °C is not observable in the TG curves of the “pure” constituents BTESE gel and calcined hydrotalcite. This shows unequivocally that both are not only chemically connected in the mixture but also that this happens via hydrogen bonding between silanol groups and active oxygen centers of the brucite layers on one side and OH groups of the brucite layers and oxygens from the silicate network on the other side following the reaction MgO-H + Si-OH → MgOSi + H_2_O. Given the basicity of hydrotalcite on the one hand and the variability of the degree of hydrolysis of the BTESE gel in dependence on pH [26] on the other hand, a variable degree of cross-linking both within BTESE siloxane rings and between siloxane rings and brucite layers could be achieved for the design of membranes with different properties.

## 4. Conclusions

This study presents a novel approach for the preparation of mixed-matrix membranes for gas separation consisting of hydrotalcite dispersed in a matrix of an organo-silica gel derived from 1,2-bis(triethoxysilyl)ethane (BTESE). The procedure includes synthesis and subsequent agent-free delamination of hydrotalcite nanoparticles. This is followed by the dispersion of the delaminated and calcined hydrotalcite in the organo-silica gel matrix. The materials were systematically examined after each preparation step by XRD, Raman, IR spectroscopy, and TG analysis. The results show substantial evidence for the successful preparation, delamination, dispersion, and calcination processes. XRD provides valuable information about the layer thickness, unit cell parameters, and size of coherent scattering domains of hydrotalcite after synthesis and every treatment step. The synthesis in methanol produces nano-sized hydrotalcite with an average of two brucite layer thicknesses. The consequent hydrolysis and delamination are proven by the lowering of the c-unit cell parameter and the formation of stacking faults along the z direction. The results from IR and Raman spectroscopy reveal the nature of the bonding mechanisms between hydrotalcite and BTESE-derived gel. These include forming a strong hydrogen bonding system between the interlayer species (OH groups, CO_3_^2−^) of hydrotalcite and siloxane oxygens on one hand and silanol active gel centers and bridging oxygens from brucite layers on the other. The interaction between ethoxide groups and hydrotalcite can also not be excluded, but further investigations are necessary to elucidate their role. These findings may give an impetus for further development of mixed-membranes containing inorganic sorption or catalytic agents combined with organo-silica networks. Part 2 of this work will present a study of all the investigated materials for their permeation and separation behavior.

## Figures and Tables

**Table 1:** Unit cell parameters and size of the coherent scattering domains of hydrotalcite in samples 1–4 refined for 3R (space group R-3m H) and 2H (P6_3_/mmc) polytypes.

XRD Results	Samples				
u.c.p.	1	2a	2b	3	4
a (Å)	3.07(1)	3.066(1)	3.14(1)	3.10(5)	3.064(1)
c (Å) 3R	25.25(1)	23.683(2)	23.557(2)	24.22(1)	24.25(1)
c (Å) 2H	16.833(7)	15.788(1)	15.705(2)	16.19(1)	16.181(7)
crystal size (nm)	1.6(1)	10.6(2)	8.42(7)	3.2(1)	3.4(1)

**Table 2:** Band assignment of the IR spectra in Figure 2.

Hydrotalcite				BTESE, HTlc-Modified Organo-Silica Membrane		
Sample	1	2a,b	3	Sample	BTESE	4
Band Assignment	Band Positions (cm^−1^)			Band Assignment	Band Positions (cm^−1^)	
ν Mg(Al)-O	630			ν Mg(Al)-O		650–700
OH libration		775		OH libration		765
ν_2_ (CO_3_)	870	860	853	ν_1_ Si-O	800	830
ν_1_ (CO_3_)	1075	1075	1075	Si–OH	950sh	909
ν_3_ (CO_3_)	1365	1365	1370	ν_3_ Si-O	1035s	1022
M,Bν_3_ (CO_3_), ν_2_ CH_3_	1400–1500		1390	ν_1_ (CO_3_)		1086
δ H_2_O	1645	1635	1635	ν_3_ Si-O	1150sh	1152
H-bonding	3000–3400	3100–3400	3200–3400	ν Si-C	1280	1270
ν_1_ OH(H_2_O)	3425	3420	3475	ν_3_ (CO_3_) 1370 M,Bν_3_ (CO_3_) 1405		
				δ H_2_O		1638
				ν-CH_2_		2890
				ν-CH_3_		2980
				H-bonding ν_1_ OH(H_2_O		3260 3370

M,Bν_3_ (CO_3_)—Monodentate, Bidentate CO_3_, sh—shoulder, s—strong band.

## Data Availability

The data that support the findings of this study are available from the corresponding author upon request.

## References

[1] Bünger L., Kurtz T., Garbev K., Stemmermann P., Stapf D. (2024). Mixed-Matrix Organo–Silica–Hydrotalcite Membrane for CO_2_ Separation Part 2: Permeation and Selectivity Study. Membranes.

[2] Hochstetter C. (1842). Untersuchung über die Zusammensetzung einiger Mineralien. J. Prakt. Chem..

[3] Mills S.J., Christy A.G., Schmitt R.T. (2016). The creation of neotypes for hydrotalcite. Mineral. Mag..

[4] Allmann R., Jepsen H.P. (1969). Die Struktur des Hydrotalkits. Neues Jb. Miner. Monat..

[5] Bellotto M., Rebours B., Clause O., Lynch J., Bazin D., Elkaim E. (1996). A reexamination of hydrotalcite crystal chemistry. J. Phys. Chem..

[6] Zhitova E., Krivovichev S., Pekov I., Greenwell H. (2019). Crystal chemistry of natural layered double hydroxides. 5. Single-crystal structure refinement of hydrotalcite, [Mg_6_Al_2_(OH)_16_](CO_3_)(H_2_O)_4_. Mineral. Mag..

[7] Evans D.G., Slade R.C.T., Duan X., Evans D.G. (2005). Structural Aspects of Layered Double Hydroxides. Layered Double Hydroxides. Structure and Bonding.

[8] Ram Reddy M.K., Xu Z.P., Lu G.Q., Diniz da Costa J.C. (2006). Layered Double Hydroxides for CO_2_ Capture: Structure Evolution and Regeneration. Ind. Eng. Chem. Res..

[9] León M., Díaz E., Bennici S., Vega A., Ordóñez S., Auroux A. (2010). Adsorption of CO_2_ on Hydrotalcite-Derived Mixed Oxides: Sorption Mechanisms and Consequences for Adsorption Irreversibility. Ind. Eng. Chem. Res..

[10] Wang Q., Tay H.H., Ng D.J.W., Chen L., Liu Y., Chang J., Zhong Z., Luo J., Borgna A. (2010). The effect of trivalent cations on the performance of Mg-M-CO_3_ layered double hydroxides for high-temperature CO_2_ capture. Chem. Sus. Chem..

[11] Bublinski M. (2017). CO2-Abtrennung aus Synthesegasen mit Hydrotalciten unter Hochtemperatur-Hochdruckbedingungen. Ph.D. Thesis.

[12] Adachi-Pagano M., Forano C., Besse J.-P. (2000). Delamination of layered double hydroxides by use of surfactants. Chem. Commun..

[13] Xie J., Khalid Z., Oh J.-M. (2022). Recent advances in the synthesis of layered double hydroxides nanosheets. B. Kor. Chem. Soc..

[14] Shirin V.A., Sankar R., Johnson A.P., Gangadharappa H., Pramod K. (2021). Advanced drug delivery applications of layered double hydroxide. J. Control. Release.

[15] Mao N., Zhou C.H., Tong D.S., Yu W.H., Lin C.C. (2017). Cynthia Lin, Exfoliation of layered double hydroxide solids into functional nanosheets. Appl. Clay Sci..

[16] Kim T.W., Sahimi M., Tsotsis T.T. (2009). The Preparation and Characterization of Hydrotalcite Thin Films. Ind. Eng. Chem. Res..

[17] Fajrina N., Yusof N., Ismail A., Jaafar J., Aziz F., Salleh W., Nordin N. (2021). MgAl-CO_3_ layered double hydroxide as potential filler in substrate layer of composite membrane for enhanced carbon dioxide separation. J. Environ. Chem. Eng..

[18] Huang N., Wang C., Chen C. (2021). Ethylene vinyl acetate copolymer/Mg-Al-layered double hydroxide nanocomposite membranes applied in CO_2_/N_2_ gas separation. Polym. Compos..

[19] Wiheeb A.D., Shakir S.W., Othman M.R. (2018). Synthesis and Characterization of Mesoporous Hydrotalcite-Alumina Membrane for Carbon Dioxide Enrichment. IOP Conf. Ser. Mater. Sci. Eng..

[20] de Vos R.M., Verweij H. (1998). High-selectivity, high-flux silica membranes for gas separation. Science.

[21] Elshof J.E.T., Klein L.C., Aparicio M., Jitianu A. (2016). Hybrid Materials for Molecular Sieves. Handbook of Sol-Gel Science and Technology, Living Reference Work.

[22] Kanezashi M., Matsugasako R., Tawarayama H., Nagasawa H., Tsuru T. (2017). Pore size tuning of sol-gel-derived triethoxysilane (TRIES) membranes for gas separation. J. Membr. Sci..

[23] Van Gestel T., Velterop F., Meulenberg W.A. (2021). Meulenberg, Zirconia-supported hybrid organosilica microporous membranes for CO_2_ separation and pervaporation. Sep. Purif. Technol..

[24] Castricum H.L., Kreiter R., van Veen H.M., Blank D.H., Vente J.F., Elshof J.E.T. (2008). High-performance hybrid pervaporation membranes with superior hydrothermal and acid stability. J. Membr. Sci..

[25] Yu L., Kanezashi M., Nagasawa H., Guo M., Moriyama N., Ito K., Tsuru T. (2019). Tailoring Ultramicroporosity To Maximize CO_2_ Transport within Pyrimidine-Bridged Organosilica Membranes. ACS Appl. Mater. Interfaces.

[26] Castricum H.L., Paradis G.G., Mittelmeijer-Hazeleger M.C., Kreiter R., Vente J.F., Elshof J.E.T. (2011). Tailoring the separation behavior of hybrid organosilica membranes by adjusting the structure of the organic bridging group. Adv. Funct. Mater..

[27] Kanezashi M., Yada K., Yoshioka T., Tsuru T. (2009). Design of silica networks for development of highly permeable hydrogen separation membranes with hydrothermal stability. J. Am. Chem. Soc..

[28] Moriyama N., Nagasawa H., Kanezashi M., Ito K., Tsuru T. (2018). Bis(triethoxysilyl)ethane (BTESE)-derived silica membranes: Pore formation mechanism and gas permeation properties. J. Sol-Gel Sci. Technol..

[29] Agirre I., Arias P.L., Castricum H.L., Creatore M., Elshof J.E.T., Paradis G.G., Ngamou P.H., van Veen H.M., Vente J.F. (2014). Hybrid organosilica membranes and processes: Status and outlook. Sep. Purif. Technol..

[30] Leroux F., Adachi-Pagano M., Intissar M., Chauvière S., Forano C., Besse J.-P. (2001). Delamination and restacking of layered double hydroxides. J. Mater. Chem..

[31] Piccinni M., Bellani S., Bianca G., Bonaccorso F. (2022). Nickel-Iron Layered Double Hydroxide Dispersions in Ethanol Stabilized by Acetate Anions. Inorg. Chem..

[32] Othman M.R., Helwani Z., Martunus, Fernando W.J.N. (2009). Synthetic hydrotalcites from different routes and their application as catalysts and gas adsorbents: A review. Appl. Organometal. Chem..

[33] Gursky J.A., Blough S.D., Luna C., Gomez C., Luevano A.N., Gardner E.A. (2006). Gardner, Particle-particle interactions between layered double hydroxide nanoparticles. J. Am. Chem. Soc..

[34] Gardner E., Huntoon K.M., Pinnavaia T.J. (2001). Direct Synthesis of Alkoxide-Intercalated Derivatives of Hydrocalcite-like Layered Double Hydroxides: Precursors for the Formation of Colloidal Layered Double Hydroxide Suspensions and Transparent Thin Films. Adv. Mater..

[35] Merz D., Dregert O., Garbev K., Stemmermann P. A reliable quantitative TA-FTIR method for cementitious material characterization. Proceedings of the GEFTA-STK-Joint Meeting on Thermal Analysis and Calorimetry.

[36] Kloprogge J.T., Wharton D., Hickey L., Frost R.L. (2002). Infrared and Raman study of interlayer anions CO_3_^2−^, NO_3_^−^, SO_4_^2−^ and ClO_4_^−^ in Mg/Al-hydrotalcite. Am. Mineral..

[37] Frost R.L., Spratt H.J., Palmer S.J. (2009). Infrared and near-infrared spectroscopic study of synthetic hydrotalcites with variable divalent/trivalent cationic ratios. Spectrochim. Acta Part A Mol. Biomol. Spectrosc..

[38] Hernandez-Moreno M.J., Ulibarri M.A., Rendon J.L., Serna C.J. (1985). IR characteristics of hydrotalcite-like compounds. Phys. Chem. Miner..

[39] Mališová M., Horňáček M., Mikulec J., Hudec P., Jorík V. (2018). FTIR study of hydrotalcite. Acta Chim. Slov..

[40] Coenen K., Gallucci F., Mezari B., Hensen E., van Sint Annaland M. (2018). An in-situ IR study on the adsorption of CO_2_ and H_2_O on hydrotalcites. J. CO2 Util..

[41] Dawes A., Mason N.J., Fraser H. (2016). Using the C-O stretch to unravel the nature of hydrogen bonding in low-temperature solid methanol-water condensates. Phys. Chem. Chem. Phys. PCCP.

[42] Plyler E.K. (1952). Infrared spectra of methanol, ethanol, and n-propanol. J. Res. Nat. Bur.Stand..

[43] Rosales-Reina B., Cruz-Quesada G., Padilla-Postigo N., Irigoyen-Razquin M., Alonso-Martínez E., López-Ramón M.V., Espinal-Viguri M., Garrido J.J. (2023). Tunability of Hybrid Silica Xerogels: Surface Chemistry and Porous Texture Based on the Aromatic Precursor. Gels.

[44] Kim Y.-H., Hwang M.S., Kim H.J., Kim J.Y., Lee Y. (2001). Infrared spectroscopy study of low-dielectric-constant fluorine-incorporated and carbon-incorporated silicon oxide films. J. Appl. Phys..

[45] Meng L., Kanezashi M., Wang J., Tsuru T. (2015). Permeation properties of BTESE–TEOS organosilica membranes and application to O_2_/SO_2_ gas separation. J. Membr. Sci..

[46] Wahab M.A., Kim I., Ha C.-S. (2004). Hybrid periodic mesoporous organosilica materials prepared from 1,2-bis (triethoxysilyl) ethane and (3-cyanopropyl) triethoxysilane. Micropor. Mesopor. Mat..

[47] Keefe C.D., Gillis E.A.L., MacDonald L. (2009). Improper Hydrogen-Bonding CH Center Dot Y Interactions in Binary Methanol Systems As Studied by FTIR and Raman Spectroscopy. J. Phys. Chem. A.

[48] Yu Y., Wang Y., Lin K., Hu N., Zhou X., Liu S. (2013). Complete Raman spectral assignment of methanol in the C-H stretching region. J. Phys. Chem. A.

[49] Ishikawa K., Fujima N., Komura H. (1985). First—order Raman scattering in MgO microcrystals. J. Appl. Phys..

[50] Slotznick S.P., Shim S.-H. (2008). In situ Raman spectroscopy measurements of MgAl_2_O_4_ spinel up to 1400 °C. Am. Mineral..

[51] Kloprogge J.T., Frost R.L. (1999). Infrared emission spectroscopic study of the thermal transformation of Mg-, Ni- and Co-hydrotalcite catalysts. Appl. Catal. A.

[52] Matsui K., Satoh H., Kyoto M. (1998). Raman Spectra of Silica Gel Prepared from Triethoxysilane and Tetraethoxysilane by the Sol-Gel Method. J. Ceram. Soc. Jpn.

[53] Kanezaki E. (1998). Effect of atomic ratio Mg/Al in layers of Mg and Al Layered double hydroxide on thermal stability of hydrotalcite-like layered structure by means of in situ high temperature powder X-ray diffraction. Mat. Res. Bull..

[54] Frost R.L., Martens W., Ding Z., Kloprogge J.T. (2003). DSC and high-resolution TG of synthesized hydrotalcites of Mg and Zn. J. Therm. Anal. Calorim..

[55] Ke X., Bernal S.A., Provis J.L. (2017). Uptake of chloride and carbonate by Mg-Al and Ca-Al layered double hydroxides in simulated pore solutions of alkali-activated slag cement. Cem. Concr. Res..

[56] Kanezashi M., Yada K., Yoshioka T., Tsuru T. (2010). Organic–inorganic hybrid silica membranes with controlled silica network size: Preparation and gas permeation characteristics. J. Membr. Sci..

